# Voluntary Forelimbs Exercise Reduces Immobilization-Induced Mechanical Hyperalgesia in the Rat Hind Paw

**DOI:** 10.1155/2021/5592992

**Published:** 2021-08-06

**Authors:** Kumiko Ishikawa, Satoshi Oga, Kyo Goto, Junya Sakamoto, Ryo Sasaki, Yuichiro Honda, Hideki Kataoka, Minoru Okita

**Affiliations:** ^1^Department of Rehabilitation, Nagasaki University Hospital, Nagasaki, Japan; ^2^Department of Physical Therapy Science, Nagasaki University Graduate School of Biomedical Sciences, Nagasaki, Japan; ^3^Department of Physical Therapy, Faculty of Rehabilitation, Kobe Gakuin University, Kobe, Japan; ^4^Department of Rehabilitation, Nagasaki Memorial Hospital, Nagasaki, Japan; ^5^Institute of Biomedical Science, Nagasaki University, Nagasaki, Japan; ^6^Department of Rehabilitation, Juzenkai Hospital, Nagasaki, Japan

## Abstract

Voluntary exercise is sufficient to protect against neuropathic pain. However, it is unclear whether voluntary exercise reduces immobilization-induced hyperalgesia. We examined the effect of voluntary forelimb exercise on immobilized-induced hyperalgesia in hind paws of rats. Wistar rats were randomly divided into the (1) both hind limbs immobilized group (IM group), (2) immobilization and exercise with nonimmobilized fore limbs group (EX group), and (3) control group. In the IM and EX groups, the bilateral ankle joints of each rat were immobilized in full plantar flexion with a plaster cast for eight weeks. In the EX group, voluntary exercise using nonimmobilized forelimbs in the running wheel was administered during the immobilization period, while hind limbs were kept immobilized (60 min/day, 5 days/week). Mechanical hyperalgesia in the hind paw was measured using a digital von Frey device every week. To investigate the abnormality of primary sensory neurons and central sensitization, the number of calcitonin gene-related peptide-positive cells in the dorsal root ganglion and the expression level of calcitonin gene-related peptide in the spinal dorsal horn were analyzed by immunohistochemical staining. Immobilization-induced mechanical hyperalgesia was inhibited in the EX group compared to the IM group at three weeks after immobilization. In the EX group, the number of calcitonin gene-related peptide-positive cells in the dorsal root ganglion and the expression level of calcitonin gene-related peptide were significantly decreased compared to those in the IM group. Our results therefore suggest that voluntary forelimb exercise during hind limb immobilization partially reduces immobilization-induced hyperalgesia by suppressing that the plastic changes of the primary sensory nerves that excessively transmit pain and increased responsiveness of nociceptive neurons in the spinal dorsal horn.

## 1. Introduction

Limb immobilization is a widely used medical treatment for injuries. However, joint immobilization causes various degenerative and atrophic changes in intact organs and tissues, including muscular disuse atrophy and joint contractures in human and animal experiments [1, 2]. Recently, previous studies have suggested that cast immobilization without injury may cause immobilization-induced hyperalgesia [3, 4]. Some studies have demonstrated that the mechanism underlying immobilization-induced hyperalgesia involves changes in skin tissue (thinning of the epidermal layer and increased peripheral nerve density), changes in the peripheral nerve (calcitonin gene-related peptide- (CGRP-) expressing cells in the dorsal root ganglia), and changes in the spinal cord with overexpression of CGRP in the dorsal horn [5, 6]. CGRP is a neurotransmitter of nociceptive primary afferents and is mainly produced in dorsal root ganglion (DRG) of small neurons with nonmyelinated axons (C-fibers) and medium-sized neurons with myelinated axons (A*δ*-fibers). It releases to the superficial (laminae I-II) and deep layers (laminae III–VI) of the dorsal horn of the spinal cord, respectively. A previous animal study [6] reported that limb immobilization with cast causes the increase in CGRP expression in both the superficial and deep layers of the dorsal horn and altered expression of CGRP in dorsal root ganglia, i.e., increased expression in medium-sized neuron and decreased expression in small-sized neuron, which is called phenotype switch. These alterations in the nervous system are thought to be involved in immobilization-induced hyperalgesia [6, 7] and that immobilization-induced hyperalgesia may shift to chronic pain when cast immobilization is prolonged [3, 4]. Therefore, interventions should be developed to prevent the development and progression of immobilization-induced hyperalgesia even when the affected limb is immobilized.

Pain relief after exercise is known as exercise-induced hypoalgesia and has been clarified in studies on humans, animals, and various pain diseases. In a previous study, forced treadmill exercise with nonimmobilized limbs partially reduced immobilization-induced hyperalgesia in rats [8]. However, forced exercise sometimes shows a response such as the chronic stress reaction, and it is often difficult to apply forced exercise clinically in practice. In contrast, voluntary exercise (small rodents freely run a running wheel) is effective for the improvement of depression and anxiety-related behavior and is considered an exercise with less stress [9, 10]. Several studies have shown that promoting voluntary physical activity in pain model animals has an analgesic effect [11, 12]. However, it is unclear whether voluntary exercise with nonimmobilized limbs can reduce immobilization-induced hyperalgesia. Thus, we examined the effect of voluntary forelimb exercise on immobilization-induced hyperalgesia in hind paws of rats.

## 2. Materials and Methods

### 2.1. Animals

Male Wistar rats (*n* = 30, aged 8 weeks) were obtained from CLEA Japan, Inc., Tokyo, Japan) and were divided into three groups: (1) both hind limbs immobilized group (IM group, *n* = 10), (2) immobilization and exercise with nonimmobilized fore limbs group (EX group, *n* = 10), and (3) control group (*n* = 10). All rats were housed in plastic cages on a 12 h light/dark cycle. Food and water were available ad libitum. The Ethics Review Committee for Animal Experimentation of Nagasaki University approved all experiments prior to their implementation (approval number: 1803291442-3).

### 2.2. Immobilization

The rats in the IM and EX groups were anesthetized with an anesthetic combination involving medetomidine (Kyoritu Pharma Co., Ltd., Tokyo, Japan), midazolam (Sandoz Pharma Co., Ltd., Tokyo, Japan), and butorphanol (Meiji Seika Pharma Co., Ltd., Tokyo, Japan). They were mixed and diluted with saline to concentrations of 0.03, 0.4, and 0.5 mg/mL, respectively. Subsequently, their bilateral ankle joints were fixed in full plantar flexion using plaster casts. The plaster cast was replaced at least every two days to prevent loosening and edema in the hind paw. The rats were able to move freely in the cage by using their upper limbs that were not immobilized. The period of cast immobilization was eight weeks.

### 2.3. Application of Wheel-Running Exercises Using Forelimbs

**Figure 1:**
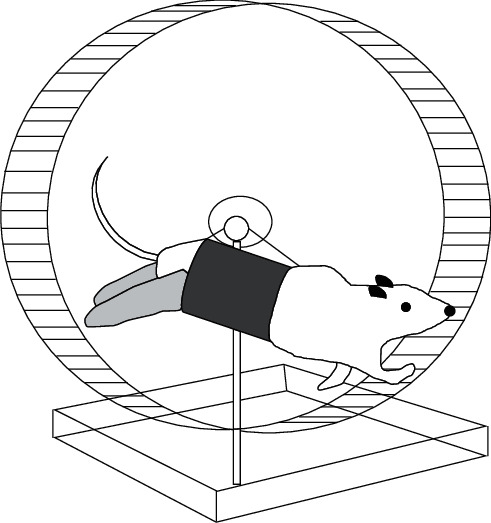
Rats in the EX group. The rats in the group are strung down from rotary shaft of the wheel with both hind limbs immobilized not to contact with the wheel and able to rotate the wheel voluntary using nonimmobilized forelimbs.

A rat wheel-running measuring device (Natsume Seisakusho Co., Ltd., Tokyo, Japan) was used for the voluntary forelimbs exercise. Rats in the EX group were individually placed in the device and strung down from the rotary shaft of the wheel with both hind limbs immobilized to avoid contact with the wheel (Figure 1). They were able to rotate the wheel voluntarily using nonimmobilized forelimbs. Before the experiment, rats in the EX group were acclimated to the exercise for a week (10 min/day). Exercise was initiated a day after immobilization and performed for 60 min/day, 5 days/week, for 8 weeks. During exercise, we recorded the fore limbs running distance and calculated the average running distance for each week.

### 2.4. The Paw Withdrawal Response (PWR)

Mechanical hyperalgesia of the hind paws was evaluated using a digital von Frey device (2391, IITC Life Science Inc., CA, USA). The animals in each group were individually placed in a homemade restrainer as described [13] to prevent immobilized hind paws from contact with the ground. All rats were allowed to acclimate for 20 min prior to testing. Subsequently, the experimenter probed the glabrous skin of the hind paw 5 times at 10 s intervals with the device and recorded the value when lifting or pulling back of the paw or vocalization as a paw withdrawal response (PWR). The average was calculated excluding the maximum and minimum values. The individual performing the evaluation was blind to the group to which the rats belonged.

### 2.5. Tissue Sampling

After eight weeks, the L4-L5 segment of the spinal cord and the associated dorsal root ganglion (DRG) of each rat were removed following transcardial perfusion with saline and 4% paraformaldehyde dissolved in 0.01 M phosphate buffer (PB) (pH 7.4). The tissue was soaked for 24 h in 10% sucrose dissolved in PB, followed by 24 h in 20% sucrose dissolved in 0.01 M PB, and then, each specimen was embedded in optimal cutting temperature (OCT) compound, frozen, and stored at −80°C.

### 2.6. Immunohistological Analysis for CGRP

Frozen spinal cord and DRG sections (10 *μ*m thick) were blocked for 30 min with 5% bovine serum albumin and goat IgG, followed by incubation with an anti-CGRP polyclonal antibody (1 : 3000; ImmunoStar Inc., Hudson, WI, USA) overnight at room temperature (22 ± 2°C). Next, they were incubated with goat anti-rabbit IgG conjugated to Texas Red® (1 : 2000, Vector Laboratories, Burlingame, CA, USA) for 1 h at room temperature. Quantitative evaluation of CGRP expression in the dorsal horn was performed using image-analysis software (NIS, Element ver. 3, Nikon Instruments Inc., Edgewood, NY, USA). Micrographs were obtained at 200x magnification, and the spinal dorsal horn was divided into the superficial (lamina I-II) and deeper (lamina III-VI) layers, according to previously described criteria [14]. Our previous study indicated how to quantitative analysis for immunostaining of CGRP in the spinal dorsal horn [15]. Briefly, the border between the superficial and the deeper layers was defined by the occurrence of transverse fibers in lamina III. The brightness of the pixels in each layer of the images was measured within the range from 0 (minimum) to 255 (maximum). Total brightness in the images was calculated, and the data were divided by the area of each layer. The fluorescence intensity was evaluated using five sections per rat. The CGRP-positive cells were counted in five unbiased images (×200) covering the entire DRG area, and the number of CGRP-positive cells per unit area (1 mm^2^) was reported as well. Subsequently, the size of the cross-sectional area of CGRP-positive neurons was calculated. Briefly, we defined negative controls that cells with a size of 2,000 *μ*m^2^ or larger that did not show CGRP expression were used as A-*β* fiber nerve cell body. Then, cells with stronger fluorescence intensity than these cells and with nuclei were defined as CGRP-positive cells in the DRG. The CGRP-positive cells were evaluated using five sections per rat. The individual performing the analysis was blind to the group to which the rats belonged.

### 2.7. Statistical Analysis

All data are presented as the mean ± SD. Differences between groups were assessed using two-way analysis of variance (ANOVA), followed by Fisher's protected least significant difference posthoc test. Immune responses were assessed using one-way ANOVA. Differences were considered significant at *p* < 0.05.

## 3. Results

### 3.1. Changes in Running Distance

**Figure 2:**
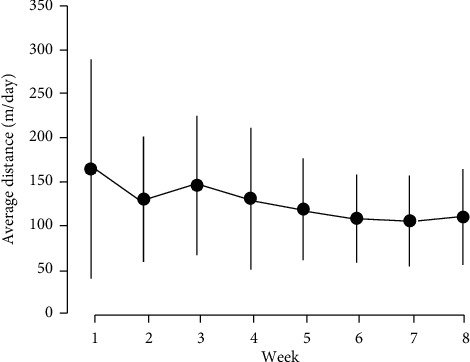
Changes in average running distance for each week in the EX group. Data are means ± SD.

In the first week, the average running distance of each rat in the EX group was the longest (164.5 ± 124.6 m) throughout the experiment. Moreover, the shortest average running distance was observed in the 7th week (105.7 ± 51.7 m). No significant differences were noted in the 8-week experimental period (Figure 2).

### 3.2. Paw Withdrawal Response

**Figure 3:**
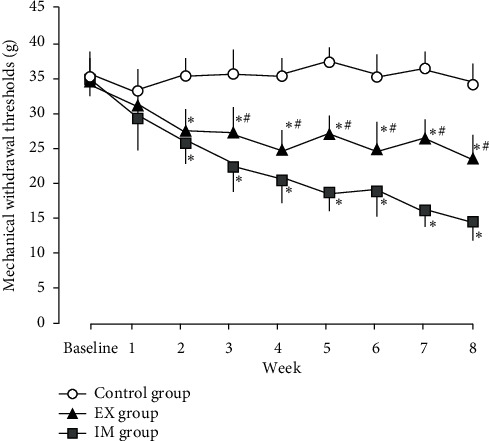
Changes over time in mechanical withdrawal thresholds (g) of hind paw. Data are mean ± SD. ^*∗*^*P* < 0.05, significantly different from the control group. ^#^*P* < 0.05, significantly different from the IM group.

At baseline and the first week, there were no significant differences between the groups. The PWR in the IM and EX groups began to decrease significantly, two weeks after immobilization compared to that in the control group. The decrease in PWR in the IM group persisted throughout the experimental period. In the EX group, the PWR was significantly higher, three weeks after immobilization compared to that in the IM group (Figure 3).

### 3.3. CGRP-Positive Neurons in the DRG

**Figure 4:**
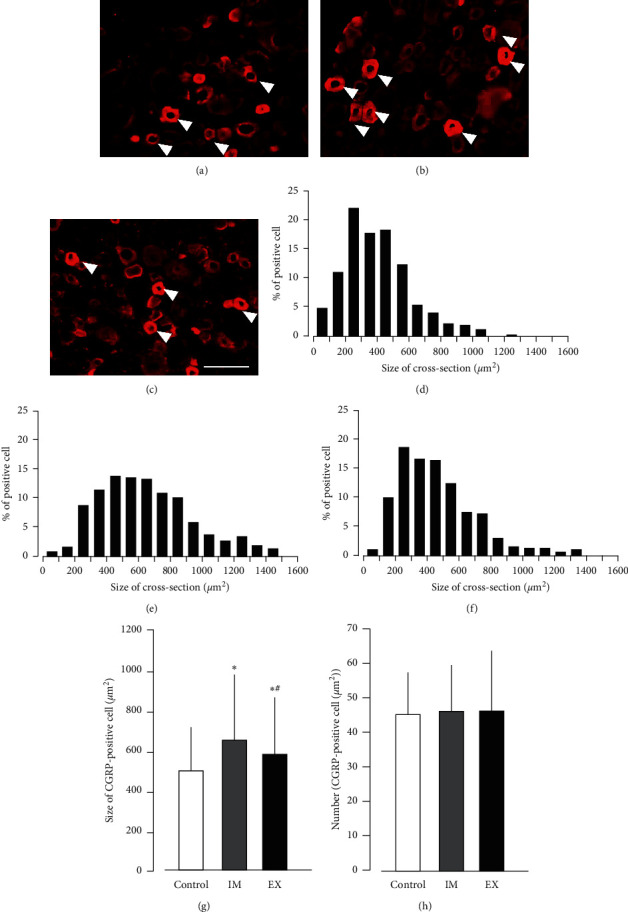
The number and cross-sectional area of calcitonin gene-related peptide- (CGRP-) positive cells in the dorsal root ganglion (DRG). Representative photomicrographs of the CGRP immunostaining of the DRG in the control (a), IM (b), and EX (c) groups. Arrowheads indicate the CGRP-positive cells in the DRG. Histograms of the size distribution of CGRP-positive DRG neurons in the control (d), IM (e), and EX groups (f). The mean of size of CGRP-positive cells (g) and number per unit area measured in the DRG (h). Data are mean ± SD. ^*∗*^*P* < 0.05, significantly different from the control group. ^#^*P* < 0.05, significantly different from the immobilization group. Scale bars = 100 *μ*m.

Compared with the control group (Figure 4(d)), the peak of the histogram shifted to the right (toward the larger size) in the IM group (Figure 4(e)), but not in the EX group (Figure 4(f)). The mean cross-sectional area of CGRP-positive cells in the DRG was significantly larger in the IM and EX groups than in the control group. In the EX group, the area of CGRP-positive neurons in the DRG was significantly smaller than that in the IM group (Figure 4(g)). Moreover, no significant differences in the number of CGRP-positive cells per unit area in the DRG were observed across the groups (Figure 4(h)).

### 3.4. Intensity of Calcitonin Gene-Related Peptide in the Spinal Dorsal Horn

**Figure 5:**
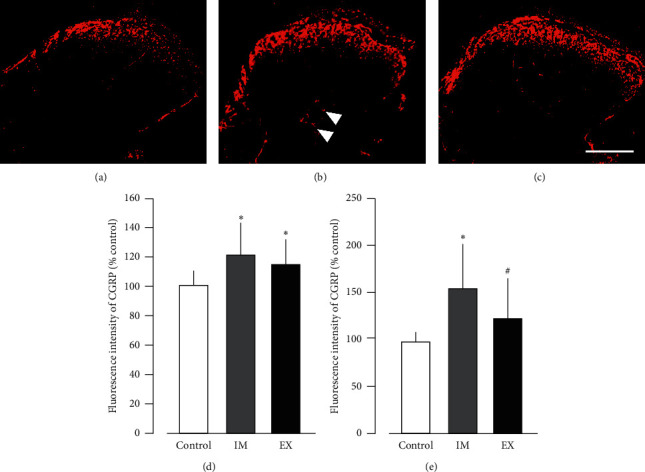
Representative photographs of CGRP immunohistochemistry in the dorsal horn at the L4-L5 in the control (a), IM (b), and EX (c) groups. Intensity of calcitonin gene-related peptide (CGRP) expression in the dorsal horn of the spinal cord. Arrowheads indicate the CGRP-positive neural fibers in the deeper layer of the dorsal horn. Percentage of fluorescence intensity of CGRP expression in the superficial layer (laminae I-II) (d) and deep layers (laminae III–VI) is calculated (e). Data are mean ± SD. ^*∗*^*P* < 0.05, significantly different from the control group. ^#^*P* < 0.05, significantly different from the immobilization group. Scale bars = 200 *μ*m.

The findings of CGRP in the spinal dorsal horn showed a markedly higher immune response in the deeper layer (laminae III–VI) of the IM group than in the control and EX groups (Figure 5(e)). In the superficial layer, CGRP expression intensity in the IM and EX groups was significantly greater than that of the control group, but no significant difference was observed between the IM and EX groups (Figure 5(d)). In contrast, regarding the expression of CGRP in the deep layer, the EX group was significantly lower than the IM group (Figure 5(e)).

## 4. Discussion

The results of this study demonstrated that voluntary forelimb exercise reduces immobilization-induced hind limb mechanical hyperalgesia and suppresses the alternations of primary sensory neurons and central sensitization in the spinal cord induced by limb immobilization.

Limb immobilization induces mechanical hyperalgesia and exacerbates inflammatory hyperalgesia. Hindlimb immobilization has been suggested to induce mechanical hyperalgesia in the paw [4, 5, 13]. In these studies, hind paw hyperalgesia was provoked after two weeks of unilateral hind limb immobilization and became prominent according to the duration of immobilization. In this study, PWR in the IM and EX groups decreased significantly after two weeks. Moreover, paw hyperalgesia in the IM group decreased continuously over the 8-week period. These results were similar to those of previous studies and suggested that the animal model in this study is valid as an immobilization-induced hyperalgesia model.

The analgesic potency of exercise has been demonstrated in animal experiments [16, 17] and clinical research [18, 19], and exercise is the recommended nonpharmacological treatment for neuropathic and inflammatory pain.

We previously examined the effect of forelimbs treadmill running exercise on immobilization-induced hyperalgesia and found that exercise suppresses immobilization-induced hyperalgesia [8]. On the other hand, there has been an increasing evidence on the effects of voluntary exercise in rodents [20, 21]. These previous studies suggested that voluntary exercise attenuated neuropathic and inflammatory pain in rodent models. In addition, promoting voluntary physical activity is suitable for clinical application because it is less stressful than the previously performed forced exercise (treadmill exercise). Senba et al. showed that a voluntary running wheel attenuates neuropathic pain in mice and that its analgesic effects were equivalent to those of forced exercise (treadmill running) [22]. Thus, to reduce pain, voluntary exercise is more effective than forced exercise. We examined the effect of voluntary forelimb exercise on immobilization-induced hyperalgesia. In the EX group, the decrease in PWR was significantly higher compared to the IM group after the third week of immobilization. These results indicate that voluntary forelimb exercise cannot inhibit the development of immobilization-induced hyperalgesia but can attenuate aggravation of hyperalgesia according to the duration of immobilization after the third week of immobilization.

In this study, wheel exercise with the forelimbs was performed by setting individual rats in a device to avoid exercising with immobilized hind limbs. This is different from previous studies [20, 21] in which wheels were placed in cages. However, the rats were not stimulated to exhibit forceful exercise of the forelimbs after installation in the device, and the movement of the rats in this study might have been performed spontaneously. Whitehead et al. described that distance traveled using a running wheel for rats during the inactive phase was stable under 100 m/h, but exceeded 100 m/h in the active phase [23]. According to their results, forelimb exercise in the EX group seems to have reached a sufficiently active level and can be interpreted as voluntary exercise.

We previously reported that unilateral hind limb immobilization alters the expression of CGRP (a neurotransmitter of the nociceptive primary afferent system) in the ipsilateral DRG [4]. There was an expression of CGRP in the cell body, which is larger than the control, where there is no change in the cell number that expresses CGRP. These findings may indicate that nonpeptidergic medium-size neurons with A-*β* fibers become peptidergic neurons and begin to produce CGRP (usually produced by small neurons with C-fibers and medium-sized neurons with A*δ*-fibers). This phenomenon may be explained by a phenotype switch of primary sensory neurons. In addition, we also reported that limb immobilization increases the expression of CGRP in the superficial (laminae I-II) and deeper layers (laminae III–VI) of the spinal dorsal horn. Notably, overexpression of CGRP in the deep layers (laminae III–VI) of the dorsal horn by limb immobilization may be related to its overexpression by medium-sized sensory neurons in the DRG. These alterations in the DRG and spinal dorsal horn are thought to be involved in the mechanism of hyperalgesia caused by limb immobilization. In this study, the histogram showing the distribution of the cross-sectional area of CGRP-positive cells in the DRG, the peak of the IM group shifted to the right compared with that of the control group, and the mean cross-sectional area in the IM group was significantly higher than that in the control group. In contrast, in the EX group, the histogram showed a similar distribution to that of the control group, and the mean cross-sectional area of CGRP-positive cells in the DRG was significantly smaller than that of the IM group. Moreover, the expression of CGRP in the deeper layer of the spinal dorsal horn in the EX group was significantly lower than that in the IM group. Hence, voluntary forelimb exercise suppressed the changes in the primary sensory nerve and spinal dorsal horn induced by limb immobilization, which may lead to attenuation of aggravation of the immobilization-induced pain.

The potential mechanisms underlying voluntary forelimb exercise-induced effects on DRG peptidergic fibers remain elusive. Recent studies have reported that endogenous cannabinoids are involved in exercise-induced hypoalgesia [24]. The endocannabinoid system consists of the endogenous cannabinoids, cannabinoid receptors, and the enzymes with regard to their synthesis and degradation [25]. This system that is widely distributed throughout the body, especially in the nervous system, regulates great numbers of physiological functions including pain and neuroinflammation [26]. A recent study demonstrated that methanandamide (cannabinoid agonist) attenuated nitroglycerin-induced CGRP increments in rat trigeminal ganglia [27]. Thus, endogenous cannabinoids may regulate the production of CGRP in peripheral nerves. Based on that finding, increase of endocannabinoids by voluntary forelimb exercise may suppress the phenotypic switch of primary sensory neurons induced by limb immobilization. Meanwhile, it is well known that exercise may facilitate upregulation of neurotrophic proteins (e.g., brain-derived neurotrophic factor, neurotrophin) in damaged pain sensory neurons [28]. A previous study demonstrated that exercises for hindlimb of spinal cord injury rats significantly increase the levels of neurotrophin-3 (NT-3) and NT-4 in lumbar and thoracic spinal cord [29]. This finding may suggest that exercise promotes repair or protect in the injured pain sensory nerves system, and it affects not only the spinal cord segment that controls exercise but also the distal segment. There may be possibility that voluntary forelimb exercises in this study directly suppress the change of expression of CGRP in medium-sized sensory neurons in DRG. It is assumed that the expression of CGRP in the deep layer of the dorsal horn of the spinal cord was inhibited by suppressing the change of the primary sensory neuron, regardless of the mechanism involved. It is necessary to clarify these points using a follow-up test in the future.

## 5. Conclusions

Voluntary forelimb exercise during hind limb immobilization partially reduces immobilization-induced hyperalgesia in rats, even if the immobilized joint was not moving. It is very interesting that it can suppress changes related to immobilization-induced hyperalgesia, such as increased expression of CGRP in DRG and spinal dorsal horn, even if it is the immobile position itself exposed to inactivity. These findings may help to highlight the importance of exercise for patients who are exposed to immobility in part of their extremities, such as during cast immobilization after fracture.

## Data Availability

The data used to support the findings of this study are included within the article.

## References

[1] Okita M., Yoshimura T., Nakano J., Motomura M., Eguchi K. (2004). Effects of reduced joint mobility on sarcomere length, collagen fibril arrangement in the endomysium, and hyaluronan in rat soleus muscle. *Journal of Muscle Research and Cell Motility*.

[2] Psatha M., Wu Z., Gammie F. M. (2012). A longitudinal MRI study of muscle atrophy during lower leg immobilization following ankle fracture. *Journal of Magnetic Resonance Imaging*.

[3] Terkelsen A. J., Bach F. W., Jensen T. S. (2008). Experimental forearm immobilization in humans induces cold and mechanical hyperalgesia. *Anesthesiology*.

[4] Hamaue Y., Nakano J., Sekino Y. (2013). Immobilization-induced hypersensitivity associated with spinal cord sensitization during cast immobilization and after cast removal in rats. *The Journal of Physiological Sciences*.

[5] Sekino Y., Nakano J., Hamaue Y. (2014). Sensory hyperinnervation and increase in NGF, TRPV1 and P2X3expression in the epidermis following cast immobilization in rats. *European Journal of Pain*.

[6] Hamaue Y., Nakano J., Sekino Y. (2015). Effects of vibration therapy on immobilization-induced hypersensitivity in rats. *Physical Therapy*.

[7] Nishigami T., Osako Y., Tanaka K. (2009). Changes in calcitonin gene-related peptide expression following joint immobilization in rats. *Neuroscience Letters*.

[8] Chuganji S., Nakano J., Sekino Y., Hamaue Y., Sakamoto J., Okita M. (2015). Hyperalgesia in an immobilized rat hindlimb: effect of treadmill exercise using non-immobilized limbs. *Neuroscience Letters*.

[9] Brown D. A., Johnson M. S., Armstrong C. J. (2007). Short-term treadmill running in the rat: what kind of stressor is it?. *Journal of Applied Physiology*.

[10] Binder E., Droste S. K., Ohl F., Reul J. M. H. M. (2004). Regular voluntary exercise reduces anxiety-related behaviour and impulsiveness in mice. *Behavioural Brain Research*.

[11] Kami K., Tajima F., Senba E., Tajima F., Senba E. (2020). Plastic changes in amygdala subregions by voluntary running contribute to exercise-induced hypoalgesia in neuropathic pain model mice. *Molecular Pain*.

[12] Brito R. G., Rasmussen L. A., Sluka K. A. (2017). Regular physical activity prevents development of chronic muscle pain through modulation of supraspinal opioid and serotonergic mechanisms. *PAIN Reports*.

[13] Nakano J., Sekino Y., Hamaue Y. (2012). Changes in hind paw epidermal thickness, peripheral nerve distribution and mechanical sensitivity after immobilization in rats. *Physiological Research*.

[14] Molander C., Xu Q., Rivero-Melian C., Grant G. (1989). Cytoarchitectonic organization of the spinal cord in the rat: II. The cervical and upper thoracic cord. *The Journal of Comparative Neurology*.

[15] Ishikawa K., Kajiwara Y., Sakamoto J. (2019). Low-intensity muscle contraction exercise following the onset of arthritis improves hyperalgesia via reduction of joint inflammation and central sensitization in the spinal cord in a rat model. *Neuroscience Letters*.

[16] Almeida C., DeMaman A., Kusuda R. (2015). Exercise therapy normalizes BDNF upregulation and glial hyperactivity in a mouse model of neuropathic pain. *Pain*.

[17] Pitcher M. H., Tarum F., Rauf I. Z., Low L. A., Bushnell C. (2017). Modest amounts of voluntary exercise reduce pain- and stress-related outcomes in a rat model of persistent hind limb inflammation. *The Journal of Pain*.

[18] Dhawan S., Andrews R., Kumar L., Wadhwa S., Shukla G. (2020). A randomized controlled trial to assess the effectiveness of muscle strengthening and balancing exercises on chemotherapy-induced peripheral neuropathic pain and quality of life among cancer patients. *Cancer Nursing*.

[19] Sveaas S. H., Bilberg A., Berg I. J. (2020). High intensity exercise for 3 months reduces disease activity in axial spondyloarthritis (axSpA): a multicentre randomised trial of 100 patients. *British Journal of Sports Medicine*.

[20] Groover A. L., Ryals J. M., Guilford B. L., Wilson N. M., Christianson J. A., Wright D. E. (2013). Exercise-mediated improvements in painful neuropathy associated with prediabetes in mice. *Pain*.

[21] Cormier J., Cone K., Lanpher J. (2017). Exercise reverses pain-related weight asymmetry and differentially modulates trabecular bone microarchitecture in a rat model of osteoarthritis. *Life Sciences*.

[22] Senba E., Kami K. (2017). A new aspect of chronic pain as a lifestyle-related disease. *Neurobiology of Pain*.

[23] Whitehead R. A., Lam N. L., Sun M. S. (2017). Chronic sciatic neuropathy in rat reduces voluntary wheel-running activity with concurrent chronic mechanical allodynia. *Anesthesia & Analgesia*.

[24] Koltyn K. F., Brellenthin A. G., Cook D. B., Sehgal N., Hillard C. (2014). Mechanisms of exercise-induced hypoalgesia. *The Journal of Pain*.

[25] Busquets Garcia A., Soria-Gomez E., Bellocchio L., Marsicano G. (2016). Cannabinoid receptor type-1: breaking the dogmas. *F1000 Research*.

[26] Katarzyna M. (2007). Direct suppression of CNS autoimmune inflammation via the cannabinoid receptor CB1 on neurons and CB2 on autoreactive T cells. *Natural Medicine*.

[27] Kilinc E., Ankarali S., Torun I. E. (2020). Receptor mechanisms mediating the anti-neuroinflammatory effects of endocannabinoid system modulation in a rat model of migraine. *European Journal of Neuroscience*.

[28] Leitzelar B. N., Koltyn K. F., Exercise, Pain N. (2021). Exercise and neuropathic pain: a general overview of preclinical and clinical research. *Sports Medicine-Open*.

[29] Côté M.-P., Azzam G. A., Lemay M. A., Zhukareva V., Houlé J. D. (2011). Activity-dependent increase in neurotrophic factors is associated with an enhanced modulation of spinal reflexes after spinal cord injury. *Journal of Neurotrauma*.

